# A Randomized Double-Blind Sham-Controlled Study of Transcranial Direct Current Stimulation for Treatment-Resistant Major Depression

**DOI:** 10.3389/fpsyt.2012.00074

**Published:** 2012-08-17

**Authors:** Daniel M. Blumberger, Lisa C. Tran, Paul B. Fitzgerald, Kate E. Hoy, Zafiris J. Daskalakis

**Affiliations:** ^1^Department of Psychiatry, Centre for Addiction and Mental Health, University of TorontoToronto, ON, Canada; ^2^Monash Alfred Psychiatry Research Centre, The Alfred and Monash University Central Clinical SchoolMelbourne, VIC, Australia

**Keywords:** depression, transcranial direct current stimulation, treatment-resistance, clinical trial

## Abstract

**Objectives:** Transcranial direct current stimulation (tDCS) has demonstrated some efficacy in treatment-resistant major depression (TRD). The majority of previous controlled studies have used anodal stimulation to the left dorsolateral prefrontal cortex (DLPFC) and a control location such as the supraorbital region for the cathode. Several open-label studies have suggested effectiveness from anodal stimulation to the left DLPFC combined with cathodal stimulation to the right DLPFC. Thus, this study evaluated the efficacy of tDCS using anodal stimulation to the left DLPFC and cathodal stimulation to the right DLPFC compared to sham tDCS. **Methods:** Subjects between the ages of 18 and 65 were recruited from a tertiary care university hospital. Twenty-four subjects with TRD and a 17-item Hamilton Rating Scale for Depression greater than 21 were randomized to receive tDCS or sham tDCS. The rates of remission were compared between the two treatment groups. **Results:** The remission rates did not differ significantly between the two groups using an intention to treat analysis. More subjects in the active tDCS group had failed a course of electroconvulsive therapy in the current depressive episode. Side effects did not differ between the two groups and in general the treatment was very well tolerated. **Conclusion:** Anodal stimulation to the left DLPFC and cathodal stimulation to the right DLPFC was not efficacious in TRD. However, a number of methodological limitations warrant caution in generalizing from this study. Ongoing, controlled studies should provide further clarification on the efficacy of this stimulation configuration in TRD. ClinicalTrials.gov Identifier: NCT01078948.

## Introduction

Major Depressive Disorder (MDD) is a highly prevalent mental illness (Kessler et al., 2003; Patten et al., 2006). Despite the vast number of pharmacological and psychotherapeutic treatments that are available, as many as 50% of patients fail to respond to treatment (Pincus and Pettit, 2001; Sackeim, 2001; Fava, 2003). In addition, the pharmacological augmentation and combination strategies frequently used in treatment-resistant depression (TRD) often increase the risk of adverse events and drug interactions (Joo et al., 2002; Dew et al., 2007; Papakostas, 2008). Electroconvulsive therapy (ECT) has demonstrated superior efficacy outcomes in TRD (Eranti et al., 2007; Lisanby, 2007). However, many patients are reluctant to engage in a trial due to stigma and the risk of cognitive adverse effects (Lisanby, 2007). The need for alternative treatment strategies to optimize outcomes for patients who experience TRD has been recognized as one of the future directions for addressing this disorder (Insel, 2006).

Transcranial direct current stimulation (tDCS) is a non-invasive and non-convulsive form of brain stimulation in which a weak, direct current (typically 1–2 mA) is applied using two surface scalp electrodes. Initial studies in animals suggested that such stimulation could elicit polarity-dependent alterations in cortical excitability and activity, with anodal stimulation increasing cortical excitability and cathodal stimulation causing cortical inhibition (Bindman et al., 1964). Furthermore, these resultant changes were not limited solely to the period of stimulation, but endured for minutes to hours afterward (Bindman et al., 1964). More recently, Nitsche and Paulus (2001) demonstrated that comparable changes occurred following tDCS directed to the human motor cortex, providing further evidence of its neuromodulatory potential.

As a result of its capacity to alter cortical activity, investigators in the 1960s began to investigate tDCS as a possible treatment for depression (Costain et al., 1964; Lippold and Redfearn, 1964; Redfearn et al., 1964); however, results were mixed, methodological differences between studies confounded results, interest in pursuing tDCS waned and the development of pharmacological antidepressant agents dominated the ensuing decades. Since the 1990s, however, research in various forms of invasive and non-invasive brain stimulation such as deep brain stimulation (DBS) and repetitive transcranial magnetic stimulation (rTMS) has been re-invigorated. A resurgence of interest may be partially a consequence of the recognition that, despite advances in pharmacotherapy, treatment-resistance remained a persistent issue in the treatment of depression (Fava, 2003; Rush et al., 2006).

In spite of renewed interest in examining tDCS as a potential treatment for major depression, its efficacy, as well as its optimal stimulation parameters, have yet to be established. A recent meta-analysis that reviewed 10 studies (six of which were randomized controlled trials) reported that compared to sham tDCS, active tDCS was more effective in reducing symptoms of depression (Kalu et al., 2012). The authors caution, though, that the small number of studies hindered their meta-analysis, many of which had limited sample sizes, eligible for inclusion. A large, randomized sham-controlled trial that used anodal stimulation over the left dorsolateral prefrontal cortex (DLPFC) and cathodal stimulation over the contralateral supraorbital region showed a significantly greater improvement in depression scores in subjects receiving active tDCS compared to sham over a 3-week controlled phase, although differences in response or remission criteria were not demonstrated. However, after an additional 3 weeks in an open-label extension phase, those subjects who had received active stimulation were significantly more likely to achieve a 50% reduction in symptoms (Loo et al., 2012).

Although the pathophysiology and etiology of major depression is complex, one hypothesis underlying a number of brain stimulation studies is that there exists a pathological aberration and imbalance in the activity of the left and right prefrontal cortices, with the left DLPFC hypoactive and right DLPFC overactive in those with depression (Baxter et al., 1989; Fitzgerald et al., 2008; Grimm et al., 2008). With the aim of ameliorating this putative imbalance between the two hemispheres, many brain stimulation studies attempt to enhance the excitability of the left DLPFC while dampening the activity of the right prefrontal cortex (Fitzgerald et al., 2006; Blumberger et al., 2011). Though there is much debate in the ECT literature regarding the efficacy of unilateral and bilateral treatment, it is clear that both forms of stimulation involve widely distributed neurobiological change as a consequence of seizure generalization (Nobler et al., 2001). A recent brain imaging study has demonstrated that tDCS can produce electrode dependent changes in regional brain activity in the prefrontal cortex (Merzagora et al., 2010). Thus, there is a rationale for directing anodal tDCS over the left DLPFC, while placing cathodal stimulation over the right DLPFC.

The optimal placement of the electrodes remains under investigation – several tDCS studies, using bilateral frontal stimulation that resulted in an improvement of depressive symptoms, have positioned the cathode over the right supraorbital region rather than over the right DLPFC (Fregni et al., 2006a; Boggio et al., 2008; Loo et al., 2010). Moreover, as regions other than the prefrontal cortices have also been implicated in depression, it may be prudent to explore the effects of alternative electrode montages on the efficacy of tDCS. Another recent open-label, pilot study used fronto-extracephalic stimulation, in which anodal stimulation was directed over the right DLPFC and cathodal stimulation was directed over the right, upper arm (Martin et al., 2011). The subjects had previously participated in a tDCS trial that delivered bifrontal stimulation and subjects experienced the two treatment groups consecutively. The authors reported a 43.8% reduction in depression scores with a more rapid response when compared to bilateral frontal stimulation.

The relationship between degree of symptom severity and treatment-resistance is intrinsic to the question of efficacy of tDCS treatment. Many earlier studies that demonstrated promising results, included individuals experiencing mild to moderate depression and did not necessitate that participants meet criteria for treatment-resistance (Fregni et al., 2006a; Boggio et al., 2008; Rigonatti et al., 2008). Several, open-label studies have suggested that left DLPFC cathodal and right DLPFC anodal tDCS may be an effective treatment configuration in more severely depressed patients (Ferrucci et al., 2009; Brunoni et al., 2011a; Dell’Osso et al., 2011). Thus, the current study was designed to determine the efficacy of tDCS providing both left and right DLPFC stimulation using anodal and cathodal stimulation respectively. We hypothesized that this electrode placement configuration would lead to greater improvement compared to sham, with a larger effect size than previous unilateral approaches with anodal stimulation of the left DLPFC and cathodal stimulation over the supraorbital region. In addition, we hypothesized that tDCS would be as tolerated as well as sham stimulation with minimal side effects.

## Methods

### Subjects

Twenty-four outpatients (20 female, 4 male; mean age 47.3 years, range 24–62) were recruited from the Mood and Anxiety, Geriatric Mental Health, and Brain Stimulation Treatment and Research programs at the Centre for Addiction and Mental Health (a tertiary university teaching hospital) as well as via referrals from physicians in Ontario, Canada. All subjects had a diagnosis of unipolar Major Depressive Disorder without psychotic features and were experiencing a Major Depressive Episode, as confirmed by the Structured Clinical Interview for the DSM-IV (SCID-IV). Subjects were required to have a score of ≥21 on the 17-item Hamilton Rating Scale for Depression (HRSD-17). Subjects were required to meet stage II criteria on the Thase Scale for treatment-resistance (failure to achieve remission or inability to tolerate two trials of an antidepressant from separate classes; Thase and Rush, 1995). Concomitant medications, such as various classes of antidepressants (e.g., selective serotonin reuptake inhibitors, tricyclic antidepressants), benzodiazepines, and antipsychotics were permitted provided that subjects had been on a stable dose of their medications for at least 4 weeks prior to entering the study and were able to maintain those stable dosages for the duration of the protocol. Subjects taking anticonvulsants were ineligible for the study, as certain agents have been found to disrupt the effects of anodal tDCS (Nitsche et al., 2003). Moreover, individuals were not included in the study if they: (i) had a DSM-IV history of substance abuse or dependence in the 6-months prior to enrolling in the study; (ii) had a concomitant, major and unstable medical, or neurologic illness; (iii) had a history of seizures; (iv) were pregnant; and/or (v) met DSM-IV criteria for borderline personality disorder or antisocial personality disorder based on the SCID for DSM-IV Axis II Disorders (SCID-II). The research ethics board at the Centre for Addiction and Mental Health approved the study and all subjects provided written, informed consent prior to commencing their involvement in the trial.

### Study design and treatment

Following completion of baseline clinical measures, subjects were randomly assigned using a computer-generated randomization list with the information stored on a centralized computer to receive either active or sham tDCS. Only the treating clinician was aware of subjects’ treatment condition. Fifteen treatments, each lasting 20 min, were administered over the course of 3 weeks (one treatment per weekday) with clinical raters and subjects blind to treatment group allocation. After receiving seven treatment sessions, subjects were assessed using the Montgomery–Asberg Depression Rating Scale (MADRS; Montgomery and Asberg, 1979) and continued with the remainder of their treatment course, whereupon they were reassessed with the full clinical rating battery and the blind was broken. During the informed consent process, subjects were told that there were two treatment conditions (i.e., active or sham stimulation) and were instructed not to discuss their treatment experiences with the clinical rater.

At the time of the study design, there was no data on the bilateral electrode placement proposed. However, we postulated that 46 patients would be required to have a 80% chance of detecting, as significant at the 5% level, a decrease in the primary outcome measure from 8 in the sham group to 15 in the active tDCS group. We planned an interim analysis at the midpoint of the trial.

### Treatment protocol

Transcranial direct current stimulation treatment was delivered using a battery-operated, constant current stimulator (CX-6650; Rolf Schneider Electronics, Germany) and transmitted by two rubber electrodes (7 cm × 5 cm = 35 cm^2^), each covered by a saline-soaked sponge and affixed to the head with a headband. The anode was directed over the left DLFPC and the cathode was placed over the right DLPFC, corresponding to electrodes F3 and F4, respectively, according to the 10–20 EEG system. Neuronavigation studies (Herwig et al., 2001) have indicated that this is a reasonably accurate method of locating the DLPFC, and it has also been used in previous tDCS studies targeting the DLPFC (Fregni et al., 2005, 2006a). In the active treatment group, stimulation was delivered at 2 mA for 20 min; sham stimulation was delivered using parameters identical to those in the active condition with the exception of the stimulator being programmed to turn off after 30 s, allowing the investigators to mimic the initial somatic sensations experienced with active tDCS, but without providing putative therapeutic benefits (Gandiga et al., 2006; Ambrus et al., 2010). In both treatment arms, the stimulator was oriented in such a way that subjects were unable to view the settings of the treatment parameters on the front panel of the machine. Subjects were permitted to make up missed treatments; however, they were not allowed to miss more than four treatments over the duration of the study.

### Clinical assessments

Experienced clinical raters blind to treatment assignment administered the following rating scales at baseline and post-treatment: the MADRS, HRSD-17 (Hamilton, 1967), the Brief Psychiatric Rating Scale (Overall and Gorharn, 1962), and the Beck Depression Inventory (BDI; Beck et al., 1961). Subjects underwent an abbreviated assessment at the trial midpoint (i.e., after seven treatments) consisting of the MADRS only.

### Outcome measures

The primary outcome for the study was change from baseline to endpoint on the HRSD-17. All subjects were assessed at baseline, at the point of early treatment termination, if possible, and after 15 treatments. Secondary outcomes included remission (score ≤7) and response (50% improvement). Other measures included change from baseline to endpoint, as well as response and remission on MADRS and BDI-II.

### Data analysis

All statistical analyses were conducted using statistical software (SPSS for Windows 15.0; SPSS Inc., Chicago, IL, USA) and the analysis was conducted on an intention to treat basis. Baseline differences in demographic and clinical variables were compared between treatment groups. Continuous variables were analyzed with one-way analysis of variance (ANOVA). Categorical variables were analyzed with a two-tailed Fisher’s exact test (for dichotomous comparisons). All procedures were two-tailed and we used a significance level set at α = 0.05 for the primary outcome. Analysis of the primary outcome was performed using repeated measures ANOVA.

## Results

### Participant flow, follow-up, and sample characteristics

**Figure 1:**
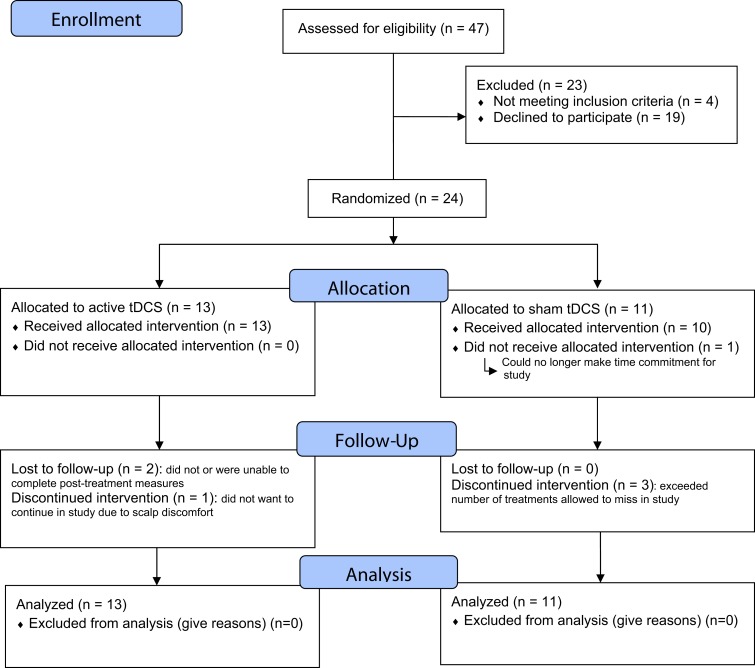
**CONSORT flow chart of the study**. The study was registered at URL: http://clinicaltrials.gov/show/NCT01078948 (ID:NCT01078948).

Of 47 patients screened, 4 did not meet eligibility criteria and 19 declined participation. A total of 24 patients were randomized (see Figure 1).

The subjects’ baseline clinical and demographic characteristics are summarized in Table 1. There were no clinically important differences between groups. Nineteen subjects were taking antidepressant medication (with or without other agents) during the trial. There were no differences in the proportion of subjects taking any of the medication classes. Six subjects in the tDCS group and two subjects in the sham group had received treatment with ECT in previous depressive episodes. Three subjects in the tDCS group and one in the sham group had failed a course of ECT during the current depressive episode. Post-treatment (week 1) data on the primary outcome measure was available for *n* = 21 subjects (87.5%). Subjects who were lost to follow-up did not differ from retained subjects on any of the baseline clinical, cognitive, or demographic variables. Nineteen subjects received all 15 treatments, of the remaining five subjects the number of missed treatments were 14, 12, 4, 1, 7 respectively. Of a total of 19 subjects who were assessed for maintenance of the blind, 14 subjects (73.7%) correctly guessed whether they received active or sham treatment: 6 (60.0%) in the active tDCS group and 8 (88.9%) in the sham group. These proportions did not differ significantly between the two groups (*p* = 0.30). The blinding of clinical raters was not assessed.

**Table 1:** **Demographic and baseline clinical characteristics**.

Characteristic	tDCS (*n* = 13)	Sham (*n* = 11)
**DEMOGRAPHICS**		
Age, years (mean, SD)	45.3 (11.6)	49.7 (9.4)
Gender, M/F	3/10	1/10
One or more medical illnesses	9 (69.2)	6 (54.5)
**DEPRESSION HISTORY**		
Recurrent episodes (%)	13 (100)	9 (81.8)
Current episode severe (%)	1 (7.7)	0 (0)
Current episode moderate (%)	12 (92.3)	11 (100)
Atypical features (%)	1 (7.7)	1 (9.1)
Melancholic features (%)	1 (7.7)	1 (9.1)
Comorbid anxiety (%)	4 (30.8)	1 (9.8)
Number of depressive episodes (mean, SD)	2.9 (2.3)	3.8 (3.7)
Duration of current episode in years (mean, SD)	4.3 (5.6)	3.4 (3.0)
Baseline HRSD (mean, SD)	24.9 (3.1)	24.1 (2.9)
Baseline MADRS (mean, SD)	31.5 (5.8)	32.0 (7.0)
Baseline BDI-II (mean, SD)	35.4 (8.1)	36.4 (6.8)
Baseline BPRS (mean, SD)	32.0 (3.3)	31.4 (3.7)
**TREATMENT HISTORY**		
SSRI (%)	2 (15.4)	2 (18.2)
SNRI (%)	7 (53.8)	2 (18.2)
Tricyclic antidepressant (%)	2 (15.4)	1 (9.1)
Mirtazapine (%)	1 (7.7)	2 (18.2)
Bupropion (%)	4 (30.8)	3 (27.3)
Antipsychotic augmentation (%)	1 (7.7)	1 (9.1)
Med combination (%)	9 (69.2)	8 (72.7)
Benzodiazepine Use (%)	6 (46.2)	2 (18.2)
History of ECT (%)	6 (46.2)	2 (18.2)
ECT failure in the current episode (%)	3 (23)	1 (9.1)
No antidepressant (%)	2 (15.4)	3 (27.3)
Number of failed antidepressant trials (mean, SD)	4.3 (2.4)	4.1 (2.2)

### Primary outcome: Change in HRSD-17

The mean post-HRSD score in the active tDCS group and sham stimulation group are shown in Table 2. There was no difference in HRSD change between the two groups (*F* = 0.063; df = 1; *p* = 0.80). The same analysis was run for all subjects who completed all 15 treatments. Similarly, there was no difference in HRSD change between the two groups (*F* = 0.30; df = 1; *p* = 0.59). None of the subjects in either group met criteria for remission on the HRSD. One subject in each group met criteria for response on the HRSD (Fisher’s exact *p* = 1.00).

**Table 2:** **Primary and secondary outcome measures (mean, SD) at baseline and post-treatment**.

	Baseline		Post-treatment	
**PRIMARY OUTCOME**				
HRSD-17 scores	tDCS (*n* = 13)	sham (*n* = 11)	tDCS (*n* = 13)	sham (*n* = 11)
	24.9 (3.1)	24.1 (2.9)	18.8 (4.77)	18.1 (5.5)
**SECONDARY OUTCOMES**				
MADRS scores	31.5 (5.8)	32.0 (7.0)	25.4 (5.2)	27.7 (6.4)
BDI-II scores	35.4 (8.1)	36.4 (6.8)	23.0 (13.8)	26.4 (8.6)

### Secondary outcome measures

#### Montgomery-asberg depression rating scale

The mean post-MADRS score in the active tDCS group and sham stimulation group are shown in Table 2. There was no difference in MADRS change between the two groups (*F* = 0.38; df = 1; *p* = 0.55). One of the subjects in the active tDCS group achieved response (Fisher’s exact *p* = 1.00) and remission criteria (Fisher’s exact *p* = 1.00) while no subjects met response or remission criteria in the sham stimulation group.

#### Beck depression inventory-II

The mean post-BDI-II score in the active tDCS group and sham stimulation group are shown in Table 2. There was no difference in BDI-II change between the two groups (*F* = 1.1; df = 1; *p* = 0.38). Two of the subjects in the active tDCS group and one in the sham stimulation group achieved remission criteria (Fisher’s exact *p* = 1.00). Three subjects in the active tDCS group and one in the sham stimulation group met criteria for response (Fisher’s exact *p* = 0.58).

### Adverse effects and tolerability

As indicated, 3/24 subjects (28%) did not complete an endpoint assessment for the primary outcome: 2/13 in the active tDCS group and 1/11 in the sham group (see Figure 1). Three subjects had an endpoint assessment but did not receive all 15 treatments as they missed too many sessions and were withdrawn. Four subjects in the sham group reported mild skin tingling. Two subjects in the active group reported mild skin tingling and two reported mild to moderate skin tingling. Three subjects in the active group reported mild headache while no subjects reported headache in the sham group. No serious adverse events were reported during the trial. One subject in the sham group withdrew due to scalp discomfort.

## Discussion

To our knowledge this is the first randomized sham-controlled trial comparing tDCS that employed anodal stimulation to the left DLPFC and cathodal stimulation to the right DLPFC. We did not find any differences between the efficacy of active and sham stimulation. Both treatment groups improved over the 3-weeks of the trial. Overall, the treatment was well tolerated with only one subject withdrawing due to scalp discomfort.

The strengths of this study included focus on inclusion of treatment-resistant subjects with stage II or higher treatment-resistance (Thase and Rush, 1995), the use of sham tDCS as a control, and an increase in the number of treatments to 15 over 3 weeks (longer than most previous treatment trials).

A number of potential limitations may explain the lack of efficacy from active tDCS in the current study. The most important limitation is the small sample size of the study. Despite the clear lack of separation between the two conditions, it is possible that differences may have been demonstrated had the study continued to its anticipated sample size of 46. Given the lack of differences between groups and the interim analysis we felt that continuing the study would not be ethical. Though we sought to include patients with treatment-resistance, the level of treatment-resistance may have been too high to observe an effect. Indeed, a third of the sample in the active stimulation group had failed a course of ECT in the current episode and nearly half had ECT in previous episodes. Though the number of subjects who failed a course of ECT in the active tDCS group was not statistically different from the number that failed a course of ECT in the sham group, the active group may have been biased toward non-response due to the small numbers in the study. Failure of ECT has generally been an exclusion criterion in other brain stimulation trials (Fregni et al., 2006b; O’Reardon et al., 2007; George et al., 2010). Future controlled trials should ensure that subjects with excessively high levels of treatment-resistance are characterized and accounted for in the randomization by stratification or excluded from the eligibility criteria. Another major limitation of the study is the high overall correct guess of treatment condition in the study. The correct guess rate calls into question the adequacy of the blinding and thus the sham control in this study. However, the sham procedure in the current study followed the directions and recommendations of previous studies (Gandiga et al., 2006; Ambrus et al., 2010). Equal numbers of subjects in both the sham and active group reported skin tingling suggesting that the sham was effective at providing a somatic sensation. However, more subjects in the active group reported headache and more intense skin tingling. It is possible that the treating technician gave non-verbal cues to subjects indicating treatment condition, however, we have no way of assessing whether this occurred. Though medication initiation was controlled in this study, the possibility remains that subjects who started an antidepressant immediately before study entry may have experienced a delayed response (i.e., greater than 4 weeks) to their antidepressant during the trial (Rush et al., 2003). However, the majority of subjects, who were taking an antidepressant, had been on stable doses of medication for longer than 8 weeks. Furthermore, the variability in the use of any antidepressant may have impacted the effect of the treatment. The use of benzodiazepines by patients may have also limited the efficacy of the treatment as this class of medication has been shown to impair the neurophysiological effects of stimulation (Nitsche et al., 2004). A greater percentage of patients in the active stimulation group were taking benzodiazepines. In addition, the patients in the active group had a longer duration of illness and had failed more medication trials. Collectively, these differences suggest that the active stimulation group were more treatment-resistant.

Notwithstanding these limitations, it is concerning that we did not demonstrate differences on any of the primary or secondary outcome measures. None of the subjects in the study met criteria for remission on the HRSD-17-item. A recent meta-analysis has also concluded that the effects of tDCS are somewhat muted (Kalu et al., 2012). A more recent randomized, double-blind, sham-controlled study in patients who had failed to respond to at least two previous trials of antidepressants from different classes did not find a difference between active left DLFPC and right supraorbital stimulation and sham stimulation of 2 weeks duration (Palm et al., 2012). Subjective ratings on secondary outcome measures, such as the Positive and Negative Affect Scale, suggested that active tDCS was associated with an increase in positive emotions and also trended toward a decrease of negative emotions (Palm et al., 2012). The largest study of tDCS in depression has recently been reported and though there was a significant difference in the change of MADRS scores, there was no difference in responders and remitters between active and sham stimulation (Loo et al., 2012). The authors suggested that longer treatment durations up to 6 weeks might be necessary to achieve clinical response with tDCS (Loo et al., 2012). We hypothesized that providing excitatory stimulation (anode) to the left DLPFC and inhibitory stimulation (cathode) to the right DLPFC would lead to improved efficacy. The theoretical rationale for this comes from the ECT literature and some previous rTMS studies showing improved efficacy with excitatory stimulation to the left DLPFC and inhibitory stimulation to the right DLPFC (Fitzgerald et al., 2006; Blumberger et al., 2011). However, recent data has not replicated the finding of improved efficacy with this stimulation pattern (Fitzgerald et al., 2012). Furthermore, it is possible that the montage of left and right DLPFC were too close together leading to shunting over the scalp. While it may be theoretically advantageous to stimulate bilaterally, the physical properties of tDCS may not be amenable to this electrode placement.

A series of three open-label trials have suggested that the stimulation technique used in this study is an effective form of tDCS in the treatment of depression (Ferrucci et al., 2009; Brunoni et al., 2011a; Dell’Osso et al., 2011). One study showed a 30% improvement in depression rating scale scores in 14 inpatients with a severe major depressive episode using twice daily treatments. Similarly, the other two studies found positive effects in both unipolar and bipolar depressed patients after 10 treatments over 5 days (Brunoni et al., 2011a; Dell’Osso et al., 2011). In contrast, we did not find this stimulation configuration to be more beneficial than sham stimulation when providing treatment once daily. However, we would caution generalizing from the current study due to the limitations identified. The design of the Sertraline vs. ELectrical Current Therapy (SELECT) tDCS trial will utilize the same stimulation parameters as the current study and should provide greater clarification regarding the efficacy of anodal stimulation to the left DLPFC and cathodal stimulation to the right DLPFC (Brunoni et al., 2011b).

## Conflict of Interest Statement

Daniel M. Blumberger receives research support for an investigator-initiated trial from Brainsway Ltd., Lisa C. Tran reports no biomedical conflicts of interest. Paul B. Fitzgerald is supported by a NHMRC Practitioner fellowship. He has received research funding from Neuronetics Inc., and equipment for investigator-initiated research from Magventure A/S and Brainsway Ltd., Zafiris J. Daskalakis receives research support for an investigator-initiated trial from Brainsway Ltd., He has received research funding from Aspect Medical Inc., and Neuronetics Inc.; he has also received a travel allowance from Pfizer.
